# When strategy must follow structure…

**DOI:** 10.3205/zma001255

**Published:** 2019-08-15

**Authors:** Sigrid Harendza

**Affiliations:** 1 Universitätsklinikum Hamburg-Eppendorf, III. Medizinische Klinik, Hamburg, Germany

## Editorial

The *Masterplan Medizinstudium 2020* prescribes structures for medical education in Germany. For instance, the selection of medical school applicants should be more targeted and undergraduate medical education itself should be more practically oriented [Masterplan Medizinstudium 2020, https://www.bmbf.de/files/2017-03-31_Masterplan%20Beschlusstext.pdf, retrieved 22.6.19]. But really, shouldn’t structure follow strategy? The basic principle that “structure follows strategy” [1], asserted by economic historian Alfred D. Chandler Jr. in the 1960s, which still applies today for the organization of large corporations, should not be irrelevant for medical school admission or the development of a more practice-based medical curriculum. The idea is that for businesses there can be no economic success if the corporate strategy, from which customers benefit in some way, is not specifically used to shape the organizational structures. In medical education, however, the structures are politically determined. For this reason, all educators active in medicine are challenged even more strongly to develop sustainable and academically solid strategies for selecting and training medical students within this framework, so that adequate healthcare can be guaranteed in Germany in the long term.

In the current joint project “stav”, funded by the Federal Ministry for Education and Research (BMBF) [Studierendenauswahl – Verbund; https://www.gesundheitsforschung-bmbf.de/de/stav-studierendenauswahl-verbund- 8229.php, retrieved 22.6.19], curricular designers and medical educators from several German universities are working to restructure the admission process for medical schools. On the one hand, a focus lies on tests that measure academic aptitude, e.g. the Aptitude Test for Medical Studies (TMS) [2], and tests that measure basic knowledge of the natural sciences, e.g. the Hamburg Natural Sciences Test (HAM-Nat) [3]. On the other hand, admission procedures addressing medical attitudes are to be developed further and piloted. An example of such a procedure is the Situational Judgement Test (SJT) of professional conduct [4]. In this procedure, applicants identify responses to specific situations by describing or evaluating the next appropriate or inappropriate step. SJTs, where behaviour is supposed to be selected how not to respond, clearly show a stronger convergent validity than SJTs, where the professional response that should be shown needs to be identified [5]. Selecting applicants who, in addition to the desired cognitive abilities, possess suitable personality traits that predict future professional behavior seems to be a good approach for further training in professional behaviour. However, scientific evidence in support of this still needs to be shown in longitudinal studies.

To develop strategies to achieve the structurally required focus on increased practice in medical education, personality characteristics and motivation also seem to be important because knowledge, skill, and attitudes are interwoven as prerequisites for competent conduct in the practice of medicine. The different aspects of physicians' roles are also represented in the learning objectives for medical competences in the National Competence Based Catalogue of Learning Objectives for Undergraduate Medical Education (NKLM) [NKLM, http://www.nklm.de, retrieved on 22.6.19]. Different facets of medical competence, for instance the acquisition of communication skills, have already been implemented in most medical curricula. A synthesis of the competences, as is required in actual medical practice, would be a desirable strategic development in medical curricula, since deficits in this regard can still be discovered in medical students. Several such deficits were exemplary identified in the BMBF-funded project ÄKHOM [Ärztliche Kompetenzen: Hamburg – Oldenburg – München; https://www.kompetenzen-im-hochschulsektor.de/aekhom/, retrieved 22.6.19], where advanced medical students participated in the resident’s role at a simulated first day of work with a 360° assessment of their medical competences. The participants received the lowest scores for the facet of competence “Structure, work planning and priorities” [6], while at the same time they felt the highest strain during the management phase of the assessment with interprofessional interactions where this competence is especially important [7]. Of particular importance for medical practice are the mastery of clinical reasoning and problem solving [8], coping with uncertainty, which is already a criterion used in the selection of medical students [9], and the development of a blame-free medical culture [10]. Within this context there are good starting points for additional strategic developments of medical curricula with a greater emphasis on practice.

How one can deal with the fact that for the development of undergraduate medical education strategy must follow structure as a result of political mandates, can be read in this issue’s position paper on *Masterplan Medizinstudium 2020* [11]. In this issue, Kunz et al. report on various feedback methods, which assist students in self-reflection and in acquiring professional attitudes, and the use of these methods in undergraduate medical education [12]. That clinical reasoning, and simultaneously a tolerance of ambiguity, can be studied with modern learning methods such as virtual patients is described by Huwendiek in his study in this issue [13]. Likewise, deliberate practice appears to be a suitable method to acquire specific competences as presented by Waechter et al. [14]. The challenges to select the right medical school applicants for the medical profession and to educate them to become competent physicians with professional attitudes and conduct, are substantial. Medical education research providing evidence, which methods should be chosen for medical school selection and how the goal of medical education with more time for practice can be reached, continues to remain indispensable for refining the selection processes and for designing medical curricula.

## Competing interests

The author declares that she has no competing interests.
